# Lyapunov Control of Quantum Systems with Impulsive Control Fields

**DOI:** 10.1155/2013/814080

**Published:** 2013-05-21

**Authors:** Wei Yang, Jitao Sun

**Affiliations:** Department of Mathematics, Tongji University, Shanghai 200092, China

## Abstract

We investigate the Lyapunov control of finite-dimensional quantum systems with impulsive control fields, where the studied quantum systems are governed by the Schrödinger equation. By three different Lyapunov functions and the invariant principle of impulsive systems, we study the convergence of quantum systems with impulsive control fields and propose new results for the mentioned quantum systems in the form of sufficient conditions. Two numerical simulations are presented to illustrate the effectiveness of the proposed control method.

## 1. Introduction 

In the last few years, because of a wide variety of applications of the quantum control theory, such as quantum chemistry, quantum information processing, and quantum electronics, considerable attention has been focused on quantum control theory, and the growing interest in this subject has been attributed to both theoretical and experimental breakthroughs ([1–9] and references therein); it indicates that quantum control has become an important area of research.

Controllability is one important part in the quantum control theory. Different definitions of controllability have been studied in [10–12], and sufficient conditions which are based on the Lie algebra of system Hamiltonian are also given. Referring to the control method, Lyapunov-based techniques are good approaches, such as implicit Lyapunov control [9, 13], Lyapunov functions based on state distance [5], average value of an imaginary mechanical quantity [14, 15], and state error [15, 16].

In [17], Turinici and Rabitz considered the wavefunction controllability method based on graph theory. When a quantum system is not wavefunction controllable with one control field, Dong and Petersen introduced the switching control method to drive the system by using two controllers to arbitrary target state based on graph theory [18]. In [19], Zhao et al. considered another switching control method of closed quantum systems, which was via the Lyapunov method.

Inspired by the switching control method, we developed the impulsive control method to drive a quantum system to a given target state. As we know, impulsive dynamical systems are a special class of dynamical systems, which exhibit continuous evolution typically described by ordinary differential equations and instantaneous state jumps or impulses. Nowadays, there has been increasing interest in the analysis and synthesis of impulsive systems, or impulsive control systems, due to their significance in both theory and applications; see [20–23] and the references therein.

By adding an impulsive control field besides the continuous control field, we apply the impulsive control method to control quantum systems. From the result in [24], when one control field contributes with given frequency, quantum systems governed by the Schrödinger equation can be described as impulsive dynamical systems.

In this paper, based on the Lyapunov method and invariant principle of impulsive systems [25], our attention is focused on the Lyapunov control of quantum systems with impulsive control fields. In Section 2, we present the quantum systems with impulsive control fields and introduce the invariant principle of impulsive systems. In Section 3, we give different control fields to drive quantum systems based on three Lyapunov functions and analyze the asymptotic stability of quantum systems with impulsive control fields. We justify the effectiveness of the proposed control fields in two simulation experiments in Section 4.

## 2. Notations and Definitions

Consider the impulsive dynamical system described by the following:

(1) $$\begin{array}{c} \dot{x}\left(t\right)=f_{c}\left(x\left(t\right)\right),\;t\in \left(\tau_{k},\tau_{k+1}\right),\\ \Delta x\left(t\right)=f_{d}\left(x\left(t\right)\right),\;t=\tau_{k}, \end{array}$$

where *x*(*t*) ∈ ℝ^*n*^ denotes the system state, *f*_*c*_(*x*) is a continuous function from ℝ^*n*^ to ℝ^*n*^, the set *E* = {*τ*_1_, *τ*_2_,…:*τ*_1_ < *τ*_2_ < ⋯} ⊂ ℝ^+^ is an unbounded, closed, and discrete subset of ℝ^+^ which denotes the set of times when jumps occur, and *f*_*d*_ : ℝ^*n*^ → ℝ^*n*^ denotes the incremental change of the state at the time *τ*_*k*_. In the *n*-dimensional complex space *ℂ* ^*n*^, we choose the most common norm $||x||:=\sqrt{x^{*}x}$, where *x* is represented as a column vector (*x*_1_, *x*_2_,…, *x*_*n*_)^*T*^ and x* denotes its conjugate transpose. Denote by *M*_*n*_(*ℂ*) the space of *n* × *n* complex matrices with an inner product (·, ·) : *M*_*n*_(*ℂ*) × *M*_*n*_(*ℂ*) → *ℂ*,

(2) $$\begin{array}{c} \left(a,b\right)=\mathrm{Tr}\left(ab\right), \end{array}$$

and the norm ||*a*||^2^ = (*a*, *a*).

Consider the following *n*-level quantum system with two control fields, and set the Plank constant *ℏ* = 1:

(3) i|ψ˙(t)〉 =(H~0+f1(t)H~1+∑k=1∞f2(t)H~2δ(t−τk))|ψ(t)〉,

where the ket |*ψ*(*t*)〉∈*ℂ* ^*n*^ represents the state vector of quantum systems, which is right continuous, and the state vector evolves on or in a sphere with radius one, and we denote the set of quantum states by *VS*_*n*_, and *δ*(·) is the Dirac impulse. Physically, two states |*ψ*_1_〉 and |*ψ*_2_〉 that differ by a phase *θ*(*t*) ∈ *R*, that is, |*ψ*_1_〉 = exp⁡ (*iθ*(*t*)) | *ψ*_2_〉, describe the same physical state in or on the sphere of *ℂ* ^*n*^. We denote the bra associated with the ket |*ψ*(*t*)〉 with 〈*ψ*(*t*)|. When the quantum system evolves freely under its own internal dynamics, that is, there is no external field implemented on the system, just the free Hamiltonian ${\tilde{H}}_{0}$ is introduced. ${\tilde{H}}_{i}\;\;(i=1,2)$ represent the interaction energy between the system and the external classical control fields *f*_*j*_(*t*)  (*j* = 1,2) and are called interaction Hamiltonian. ${\tilde{H}}_{j}\;\;(j=0,1,2)$ are all *n* × *n* self-adjoint operators in the *n*-dimensional Hilbert space *ℋ* and assumed to be time independent. In this paper, we set that the first control function *f*_1_(*t*) is a continuous function, the other one *f*_2_(*t*) only takes effect to quantum systems at the impulsive points *E*.

Multiplying both sides of (3) by −*i*, we have

(4) |ψ˙(t)〉 =(H0+f1(t)H1+∑k=1∞f2(t)H2δ(t−τk))|ψ(t)〉,

where $H_{j}=-i{\tilde{H}}_{j}\in M_{n}(C)\;\;(j=0,1,2)$, skew-Hermitian matrices.

In quantum control, the target state is usually an eigenstate of the free Hamiltonian, and we set the target state |*ψ*_*f*_〉 satisfies:

(5) $$\begin{array}{c} {\tilde{H}}_{0}|\psi_{f}\rangle =\lambda_{f}|\psi_{f}\rangle , \end{array}$$

where *λ*_*f*_ is the eigenvalue of ${\tilde{H}}_{0}$ corresponding to |*ψ*_*f*_〉.

By the same method in [24], we obtain that quantum systems (3) with impulsive control fields can be described as

(6) $$\begin{array}{c} |\dot{\psi }\left(t\right)\rangle =\left(H_{0}+f_{1}\left(t\right)H_{1}\right)|\psi \left(t\right)\rangle ,\;\;t\neq \;\tau_{k},\\ \Delta |\psi \rangle =f_{2}\left(t\right)H_{2}|\psi \left(\tau_{k}^{-}\right)\rangle ,\;t=\tau_{k}.\; \end{array}$$

When taking nontrivial geometry about states, we add a second control *ω* corresponding to *θ*(*t*) into consideration [16]: then investigate the following quantum systems

(7) |ψ˙(t)〉 =(H0+f1(t)H1+∑k=1∞f2(t)H2δ(t−τk)+ωI)|ψ(t)〉,

where *I* is the identity matrix. If the control field *f*_2_(*t*) only takes effect at the impulsive points *E*, the quantum systems with impulsive control fields are

(8) $$\begin{array}{c} |\dot{\psi }\left(t\right)\rangle =\left(H_{0}+f_{1}\left(t\right)H_{1}+\omega I\right)|\psi \left(t\right)\rangle ,\;t\neq \;\tau_{k},\\ \Delta |\psi \rangle =f_{2}\left(t\right)H_{2}|\psi \left(\tau_{k}^{-}\right)\rangle ,\;t=\tau_{k}.\; \end{array}$$

Subject to quantum systems (3) or (7), we focus on finding control fields *f*_1_(*t*) and *f*_2_(*τ*_*k*_), such that the quantum systems with impulsive control field (6) or (8) are driven to target states. Firstly, we introduce the invariant principle of impulsive systems.

**Lemma 1 (see [25])**

Consider the impulsive dynamical system (1), assume that *𝒟*_*c*_ ⊂ *𝒟* is a compact positively invariant set with respect to (1), and assume that there exists a *C* ^1^ function *V* : *𝒟*_*c*_ → ℝ such that

1. $\dot{V}(x(t))\leq 0,\;\;x\in {𝒟}_{c},\;\;t\neq \tau_{k}$;
2. *V*(*x*(*τ*_*k*_^−^) + *f*_*d*_(*x*(*τ*_*k*_^−^))) ≤ *V*(*x*(*τ*_*k*_^−^)), *x* ∈ *𝒟*_*c*_, *t* = *τ*_*k*_.

Let $G≜\{x\in {𝒟}_{c}:t\neq \tau_{k},\dot{V}(x(t))=0\}\cup \{x\in {𝒟}_{c}:t=\tau_{k},V\left(x(\tau_{k}^{-})+f_{d}(x(\tau_{k}^{-}))\right)=V(x(\tau_{k}^{-}))\}$, and let *M* ⊂ *G* denote the largest invariant set contained in *G*. If *x*_0_ ∈ *𝒟*_*c*_, then *x*(*t*) → *M* as *t* → *∞*.

## 3. Main Results

**Theorem 2**

For quantum system (6), if *H*_0_ is nondegenerate, set control fields f1(t)=K1g1(Im⁡(ei∠〈ψ(t)|ψf〉〈ψf|H~1|ψ(t)〉)) and f2(τk)=K2g2(Im⁡(ei∠〈ψ(τk-)|ψf〉〈ψf|H~2|ψ(τk-)〉)) where constants *K*_1_, *K*_2_ > 0 and the image of function *y*_*j*_ = *g*_*j*_(*x*_*j*_)  (*j* = 1,2) passes the origin of plane *x*_*j*_-*y*_*j*_ monotonically and lies in quadrant I or III, then quantum systems with impulses (6) converge to the largest invariant set *VS*_*n*_∩*E*_1_ where $E_{1}=\{|\psi \rangle :\langle \psi_{f}|{\tilde{H}}_{1}|\psi \rangle =0\}$. If all the states in *E*_1_ are equivalent to the target state |*ψ*_*f*_〉, then the systems will converge asymptotically to the target state |*ψ*_*f*_〉.

**Proof**

Choose the Lyapunov function based on the state distance

(9) $$\begin{array}{c} V\left(|\psi \left(t\right)\rangle ,t\right)=\frac{1}{2}\left(1-{\left|\langle \psi_{f}|\psi \left(t\right)\rangle \right|}^{2}\right). \end{array}$$

When *t* ≠ *τ*_*k*_,

(10) V˙1=−f1(t)Im⁡(〈ψf|H~1|ψ(t)〉〈ψ(t)|ψf〉)=−f1(t)|〈ψ(t)|ψf〉|Im⁡(ei∠〈ψ(t)|ψf〉〈ψf|H~1|ψ(t)〉),

as discussed in [15], by the control field

(11) f1(t)=K1g1(Im⁡(ei∠〈ψ(t)|ψf〉〈ψf|H~1|ψ(t)〉)),

we have

(12) V˙1(t)=−K1|〈ψ(t)|ψf〉|Im⁡(ei∠〈ψ(t)|ψf〉〈ψf|H~1|ψ(t)〉) ×g1(Im⁡(ei∠〈ψ(t)|ψf〉〈ψf|H~1|ψ(t)〉))<0 (t≠τk).

When *t* = *τ*_*k*_,

(13) V(|ψ(τk)〉,τk)  =V(|ψ(τk+)〉,τk+)  =12(1−〈ψ(τk−)|(I−f2(τk)H2)|ψf〉      ×〈ψf|(I+f2(τk)H2)|ψ(τk−)〉)  =V(|ψ(τk−)〉,τk−)−f2(τk)|〈ψ(τk−)|ψf〉|   ×Im⁡(ei∠〈ψ(τk−)|ψf〉〈ψf|H~2|ψ(τk−)〉)   −12f22(τk)〈ψ(τk−)|H~2|ψf〉〈ψf|H~2|ψ(τk−)〉,

by the control field

(14) f2(τk)=K2g2(Im⁡(ei∠〈ψ(τk−)|ψf〉〈ψf|H~2|ψ(τk−)〉)),

and $\langle \psi (\tau_{k}^{-})|{\tilde{H}}_{2}|\psi_{f}\rangle \langle \psi_{f}|{\tilde{H}}_{2}|\psi (\tau_{k}^{-})\rangle >0$, we have

(15) $$\begin{array}{c} V\left(|\psi \left(\tau_{k}\right)\rangle ,\tau_{k}\right)<V\left(|\psi \left(\tau_{k}^{-}\right)\rangle ,\tau_{k}^{-}\right), \end{array}$$

where *K*_*j*_  (*j* = 1, 2) can be chosen properly to adjust the control amplitude. If 〈*ψ*(*t*) | *ψ*_*f*_〉 = 0, or 〈*ψ*(*τ*_*k*_^−^) | *ψ*_*f*_〉 = 0, we set *∠*〈*ψ*(*t*) | *ψ*_*f*_〉 = 0°, or *∠*〈*ψ*(*τ*_*k*_^−^) | *ψ*_*f*_〉 = 0°.

By the definition of invariant set and properties of limit point, if we choose the control field *f*_1_(*t*) (11) which is the same as that in [15], the largest invariant set of quantum systems with impulses (6) is *VS*_*n*_∩*E*_1_, where $E_{1}=\{|\psi \rangle :\langle \psi_{f}|{\tilde{H}}_{1}|\psi \rangle =0\}$. From the invariant principle Lemma 1, quantum systems with impulsive control fields (6) will converge to *VS*_*n*_∩*E*_1_.

Thus, we complete the proof.

When the phase *θ* is considered, we choose the Lyapunov function based on the state error [15, 16].

**Theorem 3**

For quantum system (8), if *H*_0_ is nondegenerate, set the control fields *λ*_*f*_ + *ω* = *K*_0_ *g*_0_(*Im*⁡(〈*ψ*_*f*_ | *ψ*(*t*))), f1(t)=K1g1(Im⁡(ψf|H~1|ψ(t))), and f2(τk)=-2Im⁡(〈ψ(τk-)|H~2|ψf〉)/Tr⁡(H~24), where constants *K*_*j*_ > 0  (*j* = 0,1) and the image of function *y*_*j*_ = *g*_*j*_(*x*_*j*_) passes the origin of plane *x*_*j*_-*y*_*j*_ monotonically and lies in quadrant I or III, then quantum systems with impulses (8) converge to the largest invariant set *VS*_*n*_∩*E*_2_, where E2={|ψ〉:〈ψf|H~1|ψ〉=0,Im⁡(〈ψf|ψ〉)=0}. If all the states in *E*_2_ are equivalent to the target state |*ψ*_*f*_〉, then the systems will converge asymptotically to the target state |*ψ*_*f*_〉.

**Proof**

Choose the Lyapunov function based on the state error

(16) $$\begin{array}{c} V\left(|\psi \left(t\right)\rangle ,t\right)=\langle \psi \left(t\right)-\psi_{f}|\psi \left(t\right)-\psi_{f}\rangle . \end{array}$$

When *t* ≠ *τ*_*k*_,

(17) V˙=−(λf+ω)Im⁡(〈ψf|ψ(t)〉) −f1(t)Im⁡(〈ψf|H~1|ψ(t)〉),

and the simple control field

(18) λf+ω=K0g0(Im⁡(〈ψf|ψ(t)〉)),f1=K1g1(Im⁡(〈ψf|H~1|ψ(t)〉)),

we have

(19) V˙(t)=−K0Im⁡(〈ψf|ψ(t)〉)g0(Im⁡(〈ψf|ψ(t)〉)) −K1Im⁡(〈ψf|H~1|ψ(t)〉) ×g1(Im⁡(〈ψf|H~1|ψ(t)〉))<0 (t≠τk).

When *t* = *τ*_*k*_,

(20) V(|ψ(τk)〉,τk)=V(|ψ(τk+)〉,τk+)  =(〈ψ(τk−)|(I−f2(τk)H2)−〈ψf|)    ×((I+f2(τk)H2)|ψ(τk−)〉  −|ψf〉)    =〈ψ(τk−)−ψf|ψ(τk−)−ψf〉   +2f2(τk)Im⁡(〈ψ(τk−)|H~2|ψf〉)   +f22(τk)〈ψ(τk−)|H~22|ψ(τk−)〉,

since $\langle \psi (\tau_{k}^{-})|{\tilde{H}}_{2}^{2}|\psi (\tau_{k}^{-})\rangle \leq {||\psi (\tau_{k}^{-})||}^{2}||{\tilde{H}}_{2}^{2}||\leq \sqrt{\mathrm{Tr}({\tilde{H}}_{2}^{4})}$, by the control field f2(τk)=-2Im⁡(〈ψ(τk-)|H~2|ψf〉)/Tr⁡(H~24), we have

(21) V(|ψ(τk)〉,τk)=〈ψ(τk−)−ψf|ψ(τk−)−ψf〉 −4Im2(〈ψ(τk−)|H~2|ψf〉)Tr⁡(H~24) +4Im2(〈ψ(τk−)|H~2|ψf〉)Tr⁡(H~24) ×〈ψ(τk−)|H~22|ψ(τk−)〉≤V(|ψ(τk−)〉,τk−).

Using the control field *λ*_*f*_ + *ω*, *f*_1_(*t*) (18), the largest invariant set of quantum systems with impulsive control fields (8) is *VS*_*n*_∩*E*_2_ [15, 16], where E2={|ψ〉:〈ψf|H~1|ψ〉=0,Im⁡(〈ψf|ψ〉)=0}. From the invariant principle Lemma 1, the quantum systems with impulsive control fields (8) will converge to *VS*_*n*_∩*E*_2_.

Thus, we complete the proof.

Set that the eigenvalues of *H*_0_ are *λ*_*j*_, *j* ∈ {1,2,…, *n*}, and the corresponding eigenstates are |*ϕ*_*j*_〉, *j* ∈ {1,2,…, *n*}.

**Theorem 4**

For quantum systems with impulsive control field (6), if *H*_0_ is nondegenerate, set $f_{1}(t)=-K_{1}\langle \psi (t)|[i{\tilde{H}}_{1},Q]|\psi (t)\rangle$, and $f_{2}(\tau_{k})=-\langle \psi (\tau_{k}^{-})|[i{\tilde{H}}_{2},Q]|\psi (\tau_{k}^{-})\rangle /\sqrt{\mathrm{Tr}(Q{\tilde{H}}_{2}^{2}Q{\tilde{H}}_{2}^{2})}$, where constant *K*_1_ > 0, *Q* is a positive definite Hermitian matrix and satisfies [*H*_0_, *Q*] = 0, then quantum systems with impulsive control field (6) converge to the largest invariant set *VS*_*n*_∩*E*_3_, where $E_{3}=\{|\psi \rangle :\langle \phi_{j}|{\tilde{H}}_{1}|\phi_{k}\rangle \langle \phi_{k}|\psi \rangle \langle \psi |\phi_{j}\rangle =0$,  *j*, *k* ∈ {1,2,…, *n*}}. If all the states in *E*_3_ are equivalent to the target state |*ψ*_*f*_〉, then the systems will converge asymptotically to the target state |*ψ*_*f*_〉.

**Proof**

Choose another Lyapunov function based on the average value of an imaginary mechanical quantity

(22) $$\begin{array}{c} V\left(|\psi \left(t\right)\rangle ,t\right)=\langle \psi \left(t\right)|Q|\psi \left(t\right)\rangle . \end{array}$$

Considering the case *t* ≠ *τ*_*k*_, one has

(23) V˙(t)=〈ψ(t)|[iH~0,Q]|ψ(t)〉 +f1(t)〈ψ(t)|[iH~1,Q]|ψ(t)〉,

and since there is no relation between $\left[i{\tilde{H}}_{0},Q\right]$ and the control component, we can set $\left[i{\tilde{H}}_{0},Q\right]$ for convenience. If we choose simple and effective control field

(24) $$\begin{array}{c} f_{1}\left(t\right)=-K_{1}\langle \psi \left(t\right)|\left[i{\tilde{H}}_{1},Q\right]|\psi \left(t\right)\rangle , \end{array}$$

where *K*_1_ > 0, then $\dot{V}(t)=-K_{1}{\langle \psi (t)|[i{\tilde{H}}_{1},Q]|\psi (t)\rangle }^{2}<0$.

Since the state |*ψ*(*t*)〉 is right continuous at the impulsive points, we have

(25) V(|ψ(τk)〉,τk) =V(|ψ(τk+)〉,τk+) =〈ψ(τk−)|(I−f2(τk)H2)Q(I+f2(τk)H2)|ψ(τk−)〉 =V(|ψ(τk−)〉,τk−)+f2(τk)〈ψ(τk−)|[iH~2,Q]|ψ(τk−)〉  +f22(τk)〈ψ(τk−)|H~2QH~2|ψ(τk−)〉.

For Hermitian matrices *Q* and ${\tilde{H}}_{2}$, $\langle \psi (\tau_{k}^{-})|[i{\tilde{H}}_{2},Q]|\psi (\tau_{k}^{-})\rangle$ and $\langle \psi (\tau_{k}^{-})|{\tilde{H}}_{2}Q{\tilde{H}}_{2}|\psi (\tau_{k}^{-})\rangle$ are real numbers, and

(26) 〈ψ(τk−)|H~2QH~2|ψ(τk−)〉≤|||ψ(τk−)〉||2||H~2QH~2||  ≤Tr⁡(QH~22QH~22).

By the control function $f_{2}(\tau_{k})=-\langle \psi \left(\tau_{k}^{-}\right)|\left[i{\tilde{H}}_{2},Q\right]|\psi \left(\tau_{k}^{-}\right)\rangle /\sqrt{\mathrm{Tr}(Q{\tilde{H}}_{2}^{2}Q{\tilde{H}}_{2}^{2})}$, we have

(27) $$\begin{array}{c} V\left(|\psi \left(\tau_{k}\right)\rangle ,\tau_{k}\right)<V\left(|\psi \left(\tau_{k}^{-}\right)\rangle ,\tau_{k}^{-}\right). \end{array}$$

Using the control field *f*_1_(*t*) (24), the largest invariant set of quantum systems with impulsive control field (6) is *VS*_*n*_∩*E*_3_  [15], where $E_{3}=\{|\psi \rangle :\langle \phi_{j}|{\tilde{H}}_{1}|\phi_{k}\rangle \langle \phi_{k}|\psi \rangle \langle \psi |\phi_{j}\rangle =0$, *j*, *k* ∈ {1,2,…, *n*}}. From the invariant principle Lemma 1, the quantum systems controlled by *f*_1_(*t*), *f*_2_(*τ*_*k*_) (6) will converge to *VS*_*n*_∩*E*_3_.

Thus we complete the proof.

## 4. Illustrative Examples

 In this section, in order to illustrate the effectiveness of the proposed method in this paper, two numerical simulations have been presented for two five-level quantum systems and the fourth-order Runge-Kutta method is used to solve with time steps size 0.06.

**Example 1**

Consider the five-level quantum system with internal Hamiltonian, the first control Hamiltonian [15], and the second control Hamiltonian given as follows:

(28) H~0=(1000001.2000001.3000002000002.15),H~1=(0001100011000111100011000),H~2=(0010000011100000100101010).

Let the initial state and the target state be $|\psi_{0}\rangle ={\left(\begin{array}{ccccc} 1 & 0 & 0 & 0 & 0 \end{array}\right)}^{T}$ and $|\psi_{f}\rangle ={\left(\begin{array}{ccccc} 0 & 0 & 0 & 0 & 1 \end{array}\right)}^{T}$, respectively. The parameters are chosen as *K*_1_ = 0.15, *K*_2_ = 0.001. Seting the state $|\psi (t)\rangle ={\left(\begin{array}{ccccc} x_{1} & x_{2} & x_{3} & x_{4} & x_{5} \end{array}\right)}^{T}$, by the control fields

**Figure 1:**
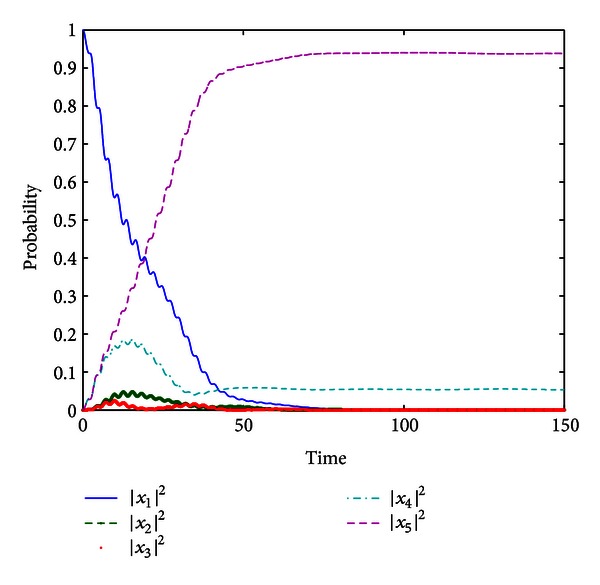
The population of the five-level system trajectory from |*ψ*_0_〉 by control fields *f*_1_(*t*) and *f*_2_(*τ*_*k*_) in Example 1.

(29) f1(t)=K1Im⁡(ei∠〈ψ(t)|ψf〉〈ψf|H~1|ψ(t)〉),f2(τk)=K2Im⁡(ei∠〈ψ(τk−)|ψf〉〈ψf|H~2|ψ(τk−)〉),

we have the simulation result shown in Figure 1. It demonstrates the control performance with impulsive control field *f*_2_(*τ*_*k*_), and the final transition probability attains about 0.94149, which excels the one (about 0.93785) in [15].

**Example 2**

Consider the five-level quantum system with internal Hamiltonian and the control Hamiltonians given as follows:

(30) H~0=(1000001.1000001.2000001.4000001.7),H~1=(0001100000000001000110010),H~2=(0010000101110000000101010).

**Figure 2:**
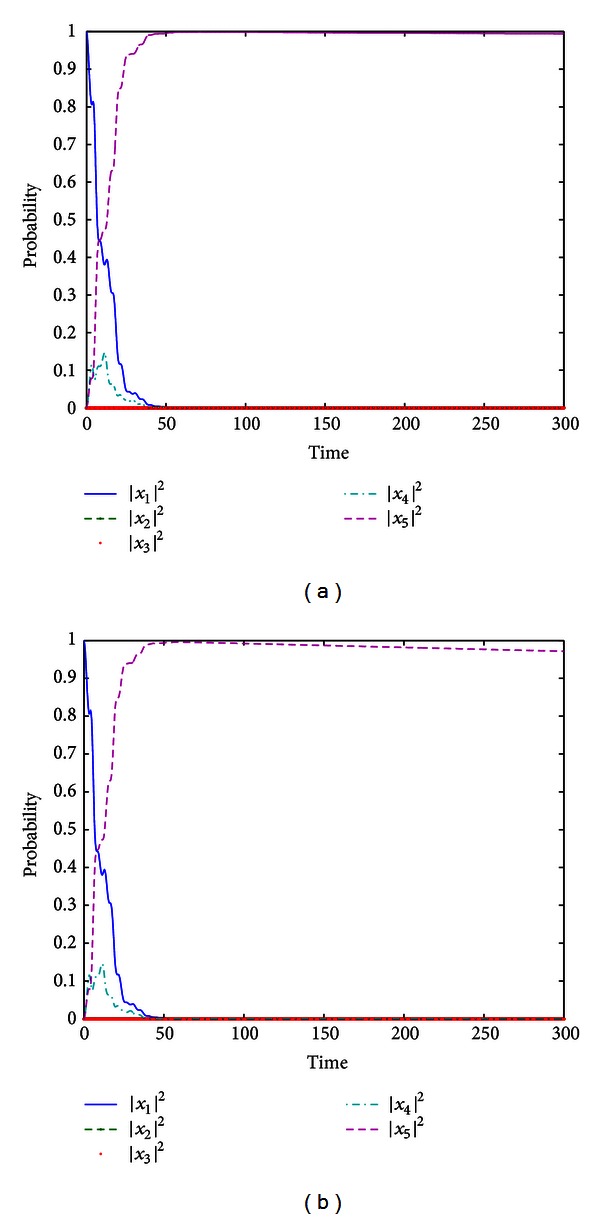
(a) The population of the five-level system trajectory from |*ψ*_0_〉 by control fields *f*_1_(*t*) and *f*_2_(*τ*_*k*_) in Example 2; (b) the population of the five-level system trajectory from |*ψ*_0_〉 without control field *f*_2_(*τ*_*k*_) in Example 2.

Let the initial state and the target state also be $|\psi_{0}\rangle ={\left(\begin{array}{ccccc} 1 & 0 & 0 & 0 & 0 \end{array}\right)}^{T}$ and $|\psi_{f}\rangle ={\left(\begin{array}{ccccc} 0 & 0 & 0 & 0 & 1 \end{array}\right)}^{T}$, respectively. The parameters are chosen as *K*_1_ = 0.15,  *K*_2_ = 0.001. Seting the state $|\psi (t)\rangle ={\left(\begin{array}{ccccc} x_{1} & x_{2} & x_{3} & x_{4} & x_{5} \end{array}\right)}^{T}$, by the same control fields in Example 1, we have the simulation results shown in Figure 2. In Figure 2(a), the population of the system with impulsive control field *f*_2_(*τ*_*k*_) is shown, and the result shown in Figure 2(b) demonstrates the control performance without impulsive control field. The quantum system whose Hamiltonians are (30) is driven to the target state |*ψ*_*f*_〉, and the final transition probability attains about 0.99942 in Figure 2(a), which is better than the one (about 0.99581) in Figure 2(b), and significantly, the control method with one impulsive control field can prevent the evolution from decaying.

## 5. Conclusion

In this paper, we have introduced the Lyapunov control method to quantum systems with impulsive control fields and given three kinds of control fields based on different Lyapunov functions. The theoretical results have been verified by numerical simulations to illustrate the effectiveness and advantages of the proposed method compared with existing results.
